# How does vertical reading affect saccade programming and lexical processing in the Roman script?

**DOI:** 10.1007/s00426-025-02154-9

**Published:** 2025-07-22

**Authors:** Zeynep G. Özkan, Jukka Hyönä, Maria Fernández-López, Manuel Perea

**Affiliations:** 1 Departamento de Metodología and ERI‑Lectura, Universitat de València, Valencia, Spain; 2 Department of Psychology, University of Turku, Turku, Finland; 3 Departament de Psicologia Bàsica and ERI-Lectura, Universitat de València, Valencia, Spain; 4 Centro de Investigación Nebrija en Cognición, Universidad Nebrija, Madrid, Spain; 5 Departamento de Metodología and ERI, Lectura. Av. Blasco Ibáñez, 21, Valencia, 46010 Spain

## Abstract

Although computational models of eye movement control in reading have focused on horizontal text layouts, vertically oriented text is also encountered in daily life in the Roman script. To examine the interplay between saccade programming and lexical processing under vertical reading in the Roman script, we manipulated (1) the layout of words in a sentence (horizontal vs. vertical) and (2) word frequency (high vs. low). In the vertical layout, the words themselves remained in standard orientation but were arranged vertically (one below the other). Eye-movement measures at the sentence level (e.g., total reading time, number of fixations) showed a cost for the vertical arrangement, primarily reflected in longer fixation durations rather than a greater number of fixations. Critically, at the target-word level, the word-frequency effect —which increased in later eye-fixation measures (gaze duration, total time)— remained similar in size across both layouts. The additive pattern of word frequency and text layout, supported by Bayes factors, suggests that slower saccade programming in the vertical format does not substantially impact lexical processing. While lexical processing can influence saccade programming, delays in saccade programming do not, in turn, alter lexical processing—a pattern that constrains current models of eye movement control in reading.

During reading, our eyes execute swift movements (i.e., saccades) as they transition from one fixated word to the next, in a delicate interplay between saccade programming and lexical processing that governs the process (see Rayner et al., 2003). In all Roman-script languages, reading proceeds horizontally from left to right within each line. Accordingly, all major computational models of eye movement control in these languages have focused on saccade programming along the horizontal axis (e.g., E-Z Reader, Reichle et al., 1998, 2003; Über-Reader, Reichle, 2021; SWIFT, Engbert et al., 2005; OB1-Reader, Snell et al., 2018).

Recently, attention has also turned to modeling *return sweeps*, the vertical saccades used when transitioning between lines of text (e.g., Parker & Slattery, 2019; Wang et al., 2024). Although horizontal saccades are the default during sentence reading in most modern print conventions, vertical eye movements are common in everyday life—for instance, when reading vertical banners, directories, newspaper headlines, or restaurant menus (see Yang, 2012). Moreover, while horizontal text is now dominant in most East-Asian writing systems, Japanese—and to a lesser extent, Chinese and Mongolian—still employs vertical reading formats, revealing the broader cross-linguistic relevance of this issue. Previous eye-movement studies in Chinese have shown that vertical reading often leads to shorter saccades and longer fixations compared to horizontal layouts (e.g., Sun et al., 1985), though these differences tend to diminish with increased experience in vertical formats (Yan et al., 2024). This adaptation appears to generalize across writing systems; for example, Hao and Yan (2025) found that Chinese–Japanese bilinguals showed cross-script transfer effects, with vertical reading experience in one writing system facilitating performance in the other.

These findings raise a broader theoretically important question: How does vertical word arrangement during reading in languages that use the Roman script affect eye movement control compared to the standard horizontal layout? This question dates back to Huey (1908), who observed that although reading speed was slower for vertical than for horizontal layouts, the difference was modest. Tinker (1955) later replicated this finding and reported that practice only marginally reduced the disadvantage associated with vertical formats.

In subsequent work, Laarni et al. (2004) compared eye movement patterns when reading sentences in horizontal versus vertical arrangements in Finnish. Using an eye-tracker and varying vertical presentation (left-justified or centered), they found slower reading speed for vertical text, mainly due to longer fixation durations rather than an increased number of fixations. Notably, this pattern suggests that certain cognitive stages (e.g., word-level identification) may remain largely unaffected by changes to the text layout, a finding that motivates more fine-grained, local analyses of reading performance.

Although the above-cited studies have shown a global slowdown in reading time under vertical arrangements, they have not addressed the similarities and differences between the factors responsible for eye movement control in the two layouts. This issue is particularly critical for examining the interplay between saccade programming and lexical processing. To fully capture these dynamics, it is necessary to go beyond global reading times and analyze local eye-movement measures on a target word embedded in sentences (see Rayner & Duffy, 1986). Computational models of eye movement control in reading (e.g., E-Z Reader, Reichle et al., 1998, 2003; Über-Reader, Reichle, 2021; SWIFT, Engbert et al., 2005; OB1-Reader, Snell et al., 2018) assume two partially interdependent components: lexical processing, which governs when to move the eyes, and saccade programming, which determines where to move them (see Rayner, 2009, for a review). Determining whether vertical reading follows the same control processes as horizontal reading is more than an empirical question—it tests the generality of these models, which were primarily developed for horizontal text. If vertical text induces systematic differences in saccade programming or lexical processing, models may need adjustments in how they handle spatial parameters and the interaction between lexical and oculomotor components. Conversely, if these processes remain similar across layouts, current models could generalize without modifications.

Since the seminal studies by Rayner and Duffy (1986) and Inhoff and Rayner (1986), the word-frequency effect (i.e., shorter fixation times for high-frequency words compared to low-frequency words) has been considered a key benchmark for testing the role of lexical processing in models of eye movement control. For instance, in the influential E-Z Reader model (Reichle et al., 2003), this effect is attributed to faster lexical processing for high-frequency words. High-frequency words complete the familiarity check (L1) and full lexical access (L2) more quickly, which triggers labile saccade programming (M1) and allows attention to shift sooner to the next word. As a result, readers spend less time fixating on high-frequency words during the initial fixation (first fixation duration), require fewer or shorter fixations (gaze duration), and make fewer regressions or rereading attempts compared to low-frequency words.

Notably, several studies have examined how a delay in lexical processing affects saccade programming, typically by comparing high- and low-frequency words embedded in sentences with atypical word formats (e.g., decorative fonts, rotated letters, handwritten, top-only, or faint contrast; see Blythe et al., 2018; Perea, 2012; Rayner et al., 2006; Slattery & Rayner, 2009; Warrington et al., 2018). All these studies reported an increase in the word-frequency effect for the atypical word formats in later eye fixation measures (e.g., gaze duration, total time), while early measures (e.g., first-fixation duration) were relatively unaffected. This pattern can be easily accommodated within the E-Z Reader model: low-frequency words, which are slower to complete the familiarity check (L1)—and even more so in an unfamiliar format—often fail to trigger labile saccade programming (M1) to the following word in time, leading to more refixations and longer first-pass fixation durations. In contrast, high-frequency words, with faster L1 completion, are less disrupted by atypical formats. This may explain why delays in lexical processing disproportionately impact low-frequency words, particularly in later reading measures.

Thus, a delay in lexical processing (due to unfamiliar word formats) induces changes in saccade programming, highlighting the interdependence between these two modules. In the present experiment, we took a complementary approach: we examined whether a delay in saccade programming due an unfamiliar layout (i.e., vertical vs. horizontal) interacts with or remains additive to lexical processing (word frequency). To this end, we embedded high- and low-frequency words in sentences presented in either a horizontal or vertical layout (see Fig. 1).

**Fig. 1:**
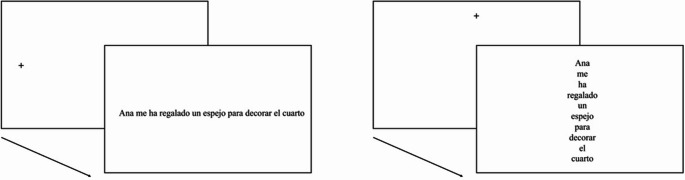
Illustration of the text layout manipulation in the experiment conducted in Spanish. The example sentence (English translation: *Ana gave me a folding screen to decorate my room*) is presented in the canonical horizontal form on the left and in the vertical arrangement on the right

This complementary approach—testing how a delay in saccade programming might cascade into lexical processing—has critical theoretical implications for current models of eye movement control in reading. As noted above, most models (E-Z Reader, SWIFT, Über-Reader, OB1-Reader) assume an interplay between lexical processing and saccade programming (e.g., unfamiliar word formats delay lexical processing and, in turn, affect saccade programming). Assessing whether a saccade-programming cost in vertical reading affects lexical processes (as captured by word frequency) allows us to determine how tightly the lexical processing and saccade programming modules are coupled in real time (i.e., whether these models can readily generalize to less typical text orientations or whether they require adjustments in some of their core assumptions).

Before presenting the two potential scenarios in the experiment, it is important to note that the word-frequency effect remains approximately the same in magnitude regardless of parafoveal availability (e.g., see Table 2 in Staub & Goddard, 2019, for review). Thus, any potential interactions with word frequency cannot be solely attributed to differences in the uptake of parafoveal information between horizontal and vertical layouts—whether due to the elliptical horizontal shape of the foveal region (McConkie & Rayner, 1975) or the closer spacing of vertically subsequent words compared to the horizontal layout.

Two potential scenarios can be considered. In the first scenario, the manipulation of saccade programming through text layout (horizontal vs. vertical) and the influence of lexical processing (word frequency: low vs. high) would operate independently, resulting in additive effects on reading times. In the second scenario, on the other hand, the two modules interact so that changes in saccade programming caused by the unfamiliar layout would affect lexical processing.

As for the first scenario, using the E-Z Reader model (Reichle et al., 2003) as a framework, one might argue that reaching the threshold for word identification in the lexical processing module (e.g., imminent word identification [L1] and full lexical access [L2]) would take more time for low-frequency words than for high-frequency words, independently of text layout. Meanwhile, saccade programming, governed by the saccade programming module (M1 and M2 stages), would take longer in the less familiar vertical layout compared to the canonical horizontal layout, regardless of word frequency. Thus, one would expect additive effects generated by the lexical processing module (i.e., a word-frequency effect) and the saccade programming module (i.e., the cost in programming the downward coordinates and executing the saccade in the less common vertical format) across the various eye fixation measures on the target word (e.g., first-fixation duration, gaze duration, total time). This outcome would suggest that slower saccade programming does not cascade into lexical processing—contrasting with the well-documented effect of slower lexical processing on saccade programming (e.g., Rayner et al., 2006).

In the second scenario, purely saccade-based delays would cascade back into lexical processing, increasing the word-frequency effect. Specifically, the increased cognitive load of programming downward saccades could hinder—or temporarily stall—covert attention shifts from the current word to the next. This disruption would delay the ongoing L2 stage (i.e., full lexical access) of the currently fixated word, particularly for low-frequency words, which already require more processing time. As a result, the word-frequency effect would be larger—especially for gaze duration and total time—when reading in a nonstandard (vertical) layout, reflecting a tighter coupling between the saccade programming and lexical processing modules than predicted by current models of eye movement control during reading.

Critically, whether the outcome is additive or interactive has direct implications for how these two modules are coordinated in real time, offering key insights into the generalizability of current models to nonstandard layouts.

## Method

### Participants

Sixty psychology students (M_age_ = 21.7, SD_age_ = 4.09) from the Universitat de València, all of them native speakers of Spanish, took part in the experiment. The sample size ensured 1800 trials per condition (60 subjects x 30 items/condition), following the guidelines proposed by Brysbaert and Stevens (2018). All participants had normal vision and were native speakers of Spanish. None of them reported having any speech or reading problems. The experiment was approved by the Ethics Committee of the Universitat de València following the Helsinki Convention. All participants signed an informed consent before the experiment and received 6 € for their participation.

### Materials

Each of the 120 experimental sentences was written in Spanish and contained either a high-frequency or a low-frequency target word (e.g., “*Mi padre tapará con un espejo/biombo la mancha de humedad*” [My father will cover with a mirror/screen the wet spot] and “*Ana me ha regalado un espejo/biombo para decorar el cuarto*” [Ana gave me a mirror/screen to decorate my room]). These sentence frames were the same as those used in previous studies (e.g., Perea & Acha, 2009; Perea et al., 2018). Sentence materials were counterbalanced using a Latin-square design across four lists, ensuring that each sentence frame appeared equally often in each of the four experimental conditions: (a) high-frequency word in the vertical layout, (b) high-frequency word in the horizontal layout, (c) low-frequency word in the vertical layout, and (d) low-frequency word in the horizontal layout. Each participant was assigned to one list and saw each sentence only once. This full counterbalancing ensured that sentence-level variability was evenly distributed across frequency and layout conditions.

As shown by Perea and Acha (2009), both high- and low-frequency target words fit naturally within the sentence frames without being predictable. Target words had an average length of 7.25 letters (range: 6–9). Low-frequency words had a mean frequency of 4.5 occurrences per million (range: 0.2–20), while high-frequency words averaged 87.3 per million (range: 23–353) (see Perea & Acha, 2009, for full details). Sentences were limited to 62 characters to fit on a single line in the horizontal layout, and target words appeared near the middle of the sentence—typically in the fourth or fifth word position.

Each participant was presented with a total of 120 sentences: 60 containing a high-frequency target word and 60 containing a low-frequency target word. Each sentence appeared in only one version (either high- or low-frequency) and in one reading orientation (vertical or horizontal). Stimuli were displayed in black 12-point Arial font on a white background. At a viewing distance of 60 cm, characters subtended approximately 0.34° of visual angle horizontally, including inter-word spacing. In the vertical layout, line spacing between words was 1 cm, corresponding to approximately 0.95° of visual angle.

### Apparatus

The experiment took place individually in a quiet, dimly lit room containing an Eyelink Portable Duo eye-tracker (SR Research Ltd, Canada) with a sampling rate of 1000 Hz, a 144 Hz 24-inch LCD Asus VG248 monitor, and a Windows-based computer running the Experiment Builder software (SR Research Ltd, Canada). Participants were seated at 60 cm of the monitor with their heads on a chin rest to reduce head movements. Only the right eye of the participant was tracked.

### Procedure

The experiment was implemented using Experiment Builder. Interest areas were defined automatically for each word based on the on-screen text layout. According to the software’s default settings, these areas were rectangular bounding boxes determined by each word’s position, font, and size parameters (see, Experiment Builder Manual, v2.3.1, SR Research, 2020). Fixations were assigned to words based on the centroid of the fixation: if the center of a fixation fell within a word’s bounding box, it was counted as a fixation on that word. This procedure was applied identically in both the horizontal and vertical layout conditions. While this method ensures consistency, vertical calibration in the EyeLink system is slightly less precise than horizontal calibration (0.73° vs. 0.56°; see Barsingerhorn et al., 2018), which may affect fixation assignment (Engbert & Nuthmann, 2008).

To familiarize the participant with reading in the vertical direction (see Tinker, 1955, for a practice effect on vertical reading) before starting the experiment and the eye tracking procedure, they participated in a training phase using the Microsoft tablet without eye-tracking. To make the vertical reading practice more engaging, participants read 42 vertically presented sentences drawn from quotes by Oscar Wilde. Before each sentence, a 500 ms fixation cross was shown at the vertical position where the sentence would begin. After reading the sentence, the participants moved to the next trial by pressing the space bar.

After training, participants placed their head on the chin rest for eye tracker calibration and validation, ensuring they were seated comfortably. A 9-point calibration procedure was performed, accepting only calibrations with an average error below 0.50° of visual angle. Upon successful validation, participants read nine practice trials. Each trial began with a drift check—top-aligned for vertical sentences and left-aligned for horizontal ones—and recalibration was performed whenever drift exceeded acceptable limits. After 30% of the sentences, a yes/no comprehension question appeared, which participants answered using the keyboard extension by pressing the ‘z’ key for “yes” and the ‘m’ key for “no.” The proportion of “yes” and “no” responses was evenly distributed (50/50). Horizontal and vertical sentences were presented in separate blocks, with block order (vertical vs. horizontal) counterbalanced across participants. A short break was provided halfway through the experiment.

### Data Analysis

The fixation durations less than 80 ms or greater than 800 ms were excluded, and fixations separated by saccades shorter than 0.16 pixels were merged automatically using Data Viewer Software (SR Research Ltd, Canada). A total of 28 trials were excluded because more than 60% of the words in the sentence were skipped or a track loss occurred; 11 of these were vertical trials, representing 0.06% of the dataset.

We analyzed the data by fitting Bayesian linear mixed models (BLMs) using the brms package (Bürkner, 2017) in R Studio. All models included the reading direction condition as a categorical fixed effect (horizontal direction coded as − 0.5, and vertical direction coded as 0.5). Word frequency was used as a categorical fixed effect (high frequency coded as − 0.5, and low frequency coded as 0.5) for local eye movement measures in the maximal random-effect structure model (Barr et al., 2013): DV = word_frequency x reading_direction + (1 + word_frequency x reading_direction| subject) + (1 + reading_direction| item).

As global processing measures, we computed the total number of fixations, the total reading time (i.e., the sum of all fixations from the onset of the first fixation to the end of the last), and the average fixation duration. We employed the Gaussian distribution family with log-transformed dependent variables for reading time and average fixation duration. For the total number of fixations, we used a negative binominal distribution, as fixation count per sentence is a discrete, overdispersed count variable (see Winter & Bürkner, 2021, for more details).

As local processing measures, we computed three early processing measures, skipping probability, first-fixation duration and gaze duration, and one later processing measure, total viewing time. For these time measures, we employed the Gaussian distribution family with log-transformed dependent variables. To fit the model for skipping rates, we used the Bernoulli distribution family. As suggested by a reviewer, we also computed the refixation rates and regression rates on the target words. For every model, we utilized 5,000 iterations, with the initial 1,000 iterations serving as a warm-up period, employing four chains. In both global and local measures, all chains in the Bayesian linear mixed-effects models successfully reached convergence, confirmed by R̂ = 1.00 for all estimates.

As one of the potential outcomes of the experiment is the presence of additive effects between the two factors (word-frequency [high, low], arrangement [vertical, horizontal]), we also computed the Bayes Factors from the Bayesian linear mixed-effects models using the bridge sampling function (Bürkner, 2017). For instance, for a given effect, if BF_01_ = 12.1, the null hypothesis would be 12.1 times more likely than the alternative hypothesis with the given set of data; in contrast a BF_01_ close to 1 (e.g., 0.9 or 1.2) indicates near equal support for both hypotheses, making the evidence inconclusive.

In addition, to ensure that the log transformation did not obscure potential interactions, we also conducted supplementary analyses using raw (untransformed) fixation durations modeled with an ex-Gaussian distribution (see Staub, 2011). These analyses yielded the same overall pattern of results: main effects of word frequency and layout, and no evidence of an interaction between the two factors. This confirms that the additive pattern observed was not an artifact of the transformation. All analyses and items can be found in the OSF repository (https://osf.io/69wt3/?view_only=3c895a96f73440f7841064339e650a1f).

## Results

The accuracy of the comprehension questions of all participants was consistently high (*M* = 94.2%, *SD* = 0.233). Fixations shorter than 80 ms and within a 16-pixel range from a longer fixation were merged into one longer fixation. Fixations beyond 80–800 ms, as well as trials with fewer than four fixations, were excluded from the analyses (1.84% of the observations). A total of 7,069 trials remained after exclusions: 1,764 in the horizontal, high-frequency condition; 1,759 in the horizontal, low-frequency condition; 1,772 in the vertical, high-frequency condition; and 1,774 in the vertical, low-frequency condition.

## Analyses of global measures

Table 1 presents the descriptive statistics of the global measures. Compared to the horizontal arrangement, the vertical layout resulted in longer total reading times (*b* = 0.16, 95% CrI[0.12, 0.19]), longer average fixation durations (*b* = 0.11, 95% CrI[0.01, 0.09]), and a greater number of fixations (*b* = 0.05, 95% CrI[0.01, 0.08]). The average saccade length was shorter in the vertical layout; however, we do not interpret this difference further, as the physical spacing between adjacent words varies across orientations, limiting the comparability of this measure (see Supplementary Materials on the OSF for further details).

**Table 1:** Global measures for reading arrangement (mean [M] and standard deviations [SD]): total reading time (in ms), average fixation duration (in ms), number of fixations, and average saccade amplitude (in *°*)

	Total reading time		Average fixation duration		Number of fixations		Average saccade amplitude (°)	
	M	SD	M	SD	M	SD	M	SD
*Reading Direction*								
Horizontal	2079	861	204	32	10.2	3.83	3.48	2.80
Vertical	2408	933	228	41	10.6	3.63	1.84	1.62

### Analyses of local measures

Table 2 provides the descriptive statistics of the local measures.

**Table 2:** Means for the local measures (standard deviations in parentheses) as a function of reading direction and target word frequency: first-fixation duration (in ms), gaze duration (in ms), total viewing time (in ms), skipping probability, refixation (in %) and regression (in %)

Reading direction		First fixation duration	Gaze duration	Total viewing time	Target word skipping (%)	Refixation rate (%)	Regression rate (%)
Horizontal							
*Word Frequency*	High	203 (64)	242 (110)	321 (192)	6.69 (0.25)	20.3 (40.2)	11.1 (4.6)
	Low	217 (75)	280 (151)	374 (234)	4.41 (0.28)	26.5 (44.1)	12.1 (5.6)
	**Effect**	**14**	**38**	**53**	**2.28**	**6.2**	**1.0**
Vertical							
*Word Frequency*	High	243 (85)	294 (134)	344 (194)	8.83 (0.21)	22.3 (41.7)	10.4 (4.8)
	Low	260 (107)	342 (193)	412 (260)	7.64 (0.27)	28.7 (45.3)	10.9 (5.2)
	**Effect**	**17**	**48**	**68**	**1.19**	**6.4**	**0.5**

## First-fixation duration

First-fixation durations on the target words were shorter for high- than low-frequency words *(*223ms vs. 238ms, respectively; *b* = 0.06, 95% CrI[0.03, 0.08]) and in the horizontal than vertical arrangement (210ms vs. 252ms, respectively; *b* = 0.17, 95% CrI[0.14, 0.21]). Critically, the size of the word-frequency effect was similar in the horizontal and vertical dispositions, 14ms vs. 17ms, respectively (interaction: *b* = −0.01, 95% CrI[−0.04, 0.02], *BF*_*01*_ = 99.8).

## Gaze duration

Gaze durations were shorter for high-frequency than for low-frequency words (268ms vs. 311ms, respectively; *b* = 0.11, 95% CrI[0.07, 0.16]), and for words in the horizontal compared to the vertical arrangement (261ms vs. 319ms, respectively; *b* = 0.20, 95% CrI[0.16, 0.24]). Again, the effect of the two factors combined additively, 38ms vs. 48ms, respectively (interaction; *b* = −0.01, 95% CrI[−0.05, 0.04], *BF*_*01*_ = 41.6).

Notably, the reading cost associated with the vertical layout in gaze durations was primarily driven by longer fixation durations rather than by an increased number of fixations. As expected, refixation rates were higher for low-frequency words (*b* = 0.34, 95% CrI [0.11, 0.58]; see Table 2 for descriptive values); however, we found no evidence in favor of an effect of layout or an interaction between the two factors (*b* = 0.16, 95% CrI [–0.10, 0.42] and *b* = − 0.02, 95% CrI [–0.31, 0.27], respectively).

## Total viewing time

The pattern mirrored that of the other measures: a word-frequency effect (333ms vs. 393ms, for high and low-frequency words, respectively; *b* = 0.14, 95% CrI[0.08, 0.20]), a reading cost for the vertical format (348ms vs. 378ms, for the words in the horizontal and vertical layouts, respectively; *b* = 0.14, 95% CrI[0.05, 0.14]), and no signs for an interaction—the word-frequency effect was 53ms vs. 68ms in the horizontal and vertical layout, respectively; *b* = 0.01, 95% CrI[−0.05, 0.07], *BF*_*01*_ = 47.3).

Of note, the longer total viewing times observed in the vertical layout were not driven by a greater number of regressions to the target words. Regression-in rates differed by only about 1% point and were actually slightly lower in the vertical layout; they were also largely unaffected by word frequency (see Table 2 for descriptive statistics). Accordingly, the model results showed a small effect of layout (*b* = − 0.09, 95% CrI [–0.13, − 0.04]), with no evidence of an effect of word frequency (*b* = 0.03, 95% CrI [–0.03, 0.10]) or an interaction between the two factors (*b* = − 0.03, 95% CrI [–0.11, 0.05]).

## Skipping probability

High-frequency words were skipped more often than low-frequency words (7.8% vs. 6.0%; *b* = −0.39, 95% CrI[−0.73, −0.04]). Moreover, words were skipped more often in the vertical than in the horizontal arrangement (8.2% vs. 5.6%; *b* = 0.82, 95% CrI[0.39, 1.26]). No clear effect for an interaction was observed (*b* = 0.40, 95% CrI[−0.15, 0.95], *BF*_*01*_ = 0.9).

## Discussion

In the present experiment, we examined the interplay between saccade programming and lexical processing during sentence reading in Spanish, a language that uses the Roman script. Specifically, we tested whether the reading cost imposed by a nonstandard vertical arrangement—which requires downward rather than rightward saccades—has an additive or an interactive effect with lexical processing, operationalized here by comparing the recognition of low- and high-frequency words.

At a global level, the sentence-level measures revealed that readers were faster with the horizontal arrangement than with the vertical arrangement (see Laarni et al., 2004; Tinker, 1955). As in Laarni et al.’s (2004) experiment, the primary cost of reading vertically oriented text emerged in fixation durations rather than in the number of fixations.

Turning to the local analyses, we observed a sizeable layout effect: target words in the vertical arrangement produced longer fixation times (first-fixation duration, gaze duration, total time) compared to those in the horizontal arrangement. Simultaneously, we replicated the well-established word-frequency effect (see Rayner et al., 2004; Rayner et al., 2006; White, 2008) in all eye-fixation measures. Crucially, the magnitude of this frequency effect was almost identical in both layouts, with Bayes factors supporting an additive pattern. Taken together, these findings indicate that (1) lexical processing remains essentially unaffected by whether words are presented horizontally or vertically (i.e., the word-frequency effect was of similar magnitude in both layouts), and (2) the vertical layout adds a cost to saccade programming, presumably because generating downward saccades introduces atypical landing coordinates.

The additive pattern found in the experiment suggests that saccade programming and lexical processing operate relatively independently during sentence reading (see Vainio et al., 2009, for a dissociation of saccade programming in terms of landing position and predictability). This functional dissociation aligns well with the assumptions of the E-Z Reader model (Reichle et al., 2003, 2009), which separates the temporal (“when”) and spatial (“where”) elements of eye-movement control into two modules: lexical processing and saccade programming, each with early (L1, M1) and late (L2, M2) stages. In its most recent version (E-Z Reader 10; Reichle et al., 2012), the model posits that attention shifts upon completing full lexical access (L2), canceling any pending labile saccade programs (M1) and initiating a familiarity check (L1) for the next parafoveal word. The additive effect between word frequency and text layout aligns with this account: word frequency primarily influences lexical processing, whereas layout primarily affects saccade programming. This pattern highlights an asymmetry—while delays in lexical processing modulate saccade programming, delays in saccade programming do not impact lexical processing. This supports the view that these two components, though interconnected, function with a degree of independence.^1^

Consistent with this view, disruptions to lexical processing caused by atypical word formats (e.g., rotated letters, Blythe et al., 2018; handwritten words, Perea et al., 2018; faint contrast, Warrington et al., 2018) lead to increased refixations and regressions, particularly for low-frequency words, thus increasing the word-frequency effect in gaze duration and total time. In contrast, disruptions in saccade programming due to text layout do not alter lexical processing. This dissociation fits well with the E-Z Reader model, in which lexical processing determines when to move the eyes, while the spatial programming of saccades determines where to move them—with limited influence of saccade targeting errors on ongoing lexical processing.

A closer look at the skipping pattern provides additional support for this interpretation. The slightly higher skipping rate observed in the vertical layout—about 2.7% points higher than in the horizontal layout—might, at first glance, suggest enhanced parafoveal processing. However, this explanation seems unlikely given that skipping was not modulated by word frequency (i.e., an index of lexical processing). Instead, the pattern is more plausibly explained by oculomotor factors such as mislocated fixations (Engbert & Nuthmann, 2008), whereby the eyes land on a word other than the intended target due to saccade execution error. Although the vertical layout in our experiment included sufficient interword spacing (1 cm), vertically aligned text may still impose unique demands on saccade planning. Unlike the typical horizontal reading format in Latin-based scripts, vertical reading involves a shift in saccade direction and saccade planning, which may increase variability in targeting. Even in the absence of spatial crowding, such variability could lead to fixations being assigned to the following word, functionally mimicking an “overshoot”. Thus, the increased skipping rate in the vertical condition is unlikely to reflect faster lexical access, but rather subtle oculomotor differences introduced by the change in layout. This interpretation is further supported by the broader additive pattern of word frequency and layout effects observed across all reading measures.

Beyond these theoretical implications, comparing horizontal and vertical layouts opens several lines of research into how saccade programming and lexical processing interact. Future work could investigate developmental trajectories or examine differences in the perceptual span and the availability of parafoveal information across layouts. While Pan et al. (2022) demonstrated that readers of Chinese can extract semantic preview information from vertically presented text, their findings are based on a logographic script with a long-standing vertical reading tradition. By contrast, AlJassmi et al. (2025) examined parafoveal processing in a Roman-based script (English) by rotating the text 90°, and found no preview benefit for vertically presented sentences; however, rotating both the text and the words themselves may have introduced spatial processing demands that are not characteristic of natural vertical reading (see Fernández-López et al., 2021). To more precisely isolate the effect of layout, future work should examine parafoveal processing in alphabetic languages using vertically formatted text without rotating letters or words. This approach preserves the native spatial structure of upcoming words in the parafovea, allowing a cleaner test of whether vertical orientation alone disrupts lexical preprocessing during reading.

Notably, the vertical layout may also have an applied dimension, especially for individuals with right-sided hemianopia, who lack visual input from the right visual field, thereby face challenges when reading from left to right. Recently, Kuester-Gruber et al. (2024) showed that vertical text arrangements—via 90° rotated sentences—can facilitate reading in this population. The present layout—horizontally oriented words arranged from top to bottom—may offer an added advantage, as it does not require readers to adjust to rotated text (see AlJassmi et al., 2025). To handle long words, hyphenation may be used to split them across lines (Laarni et al., 2004). Whether this vertical configuration is more useful than the rotated layout in clinical contexts remains an open question.

In conclusion, the present experiment provides the first demonstration of how word arrangement in a sentence impacts eye movement control in the Roman script. While vertical reading requires longer fixation durations than horizontal reading, it does not substantially increase the total number of fixations per sentence. Furthermore, the effect of text layout is additive with word frequency, suggesting that while lexical processing can influence saccade programming, saccade programming does not, in turn, alter lexical processing. These findings support the generalizability of current models of eye movement control in reading to nonstandard text orientations, without requiring fundamental changes to their underlying architecture.

## Data Availability

All data, materials and code used for all analyses, plots, and supplementary materials are available on OSF at https://osf.io/69wt3/?view_only=3c895a96f73440f7841064339e650a1f.

## Footnotes

1 One could argue that besides the oculomotor system taking longer to program or execute the downward saccade in sentence reading, the vertical layout may potentially lead to fixations closer to the optimal viewing position (OVP) (Rayner, 1979). The OVP is primarily influenced by low-level, non-linguistic factors such as word length and spacing between words; in our case, the consistent spacing in the vertical layout may provide a visual cue indicating where the center of the word is. This would help readers fixate on the OVP and reduce the refixations needed to compensate for a landing position farther from the center of the word (see Rayner et al., 2001). This possibility was discussed in the Supplementary materials available at the OSF link.
